# Discriminable Sensing Response Behavior to Homogeneous Gases Based on n-ZnO/p-NiO Composites

**DOI:** 10.3390/nano10040785

**Published:** 2020-04-20

**Authors:** Wen-Dong Zhou, Davoud Dastan, Jing Li, Xi-Tao Yin, Qi Wang

**Affiliations:** 1 School of Chemical Engineering, University of Science and Technology Liaoning, Anshan 114051, China; zhouwendong1986@163.com; 2 School of Materials Science and Engineering, Georgia Institute of Technology, Atlanta, GA 30332, USA; 3 The Key Laboratory of Chemical Metallurgy Engineering of Liaoning Province, University of Science and Technology Liaoning, Anshan 114051, China; Lijing_as321@163.com

**Keywords:** gas sensors, homogenous gases, discriminable response, selectivity, ZnO–NiO composite

## Abstract

Metal oxide semiconductor (MOS) gas sensors have the advantages of high sensitivity, short response-recovery time and long-term stability. However, the shortcoming of poor discriminability of homogeneous gases limits their applications in gas sensors. It is well-known that the MOS materials have similar gas sensing responses to homogeneous gases such as CO and H_2_, so it is difficult for these gas sensors to distinguish the two gases. In this paper, simple sol–gel method was employed to obtain the ZnO–*x*NiO composites. Gas sensing performance results illustrated that the gas sensing properties of composites with *x* > 0.425 showed a p-type response to both CO and H_2_, while the gas sensing properties of composites with *x* < 0.425 showed an n-type response to both CO and H_2_. However, it was interesting that ZnO–0.425NiO showed a p-type response to CO but an discriminable response (n-type) to H_2_, which indicated that modulating the p-type or n-type semiconductor concentration in p-n composites could be an effective method with which to improve the discriminability of this type of gas sensor regarding CO and H_2_. The phenomenon of the special gas sensing behavior of ZnO–0.425NiO was explained based on the experimental observations and a range of characterization techniques, including XRD, HRTEM and XPS, in detail.

## 1. Introduction

Various gases, including blast furnace gas, coke oven gas, water gas and other secondary gases are used as reducing agents and fuels in metallurgical and chemical production. It is well-known that CO and H_2_ can be used as reactants for chemical and metallurgical production. H_2_ is an explosive and flammable gas [1,2], while CO is a toxic gas [3,4]. The leakages of those gases during the process of storage and transportation lead to some security issues [5]. Furthermore, it is difficult to differentiate these two gases, as they are colorless, odorless, homogeneous and reducing gases. Therefore, it is necessary to design and fabricate sensors that can accurately identify whether the leaking gas is CO or H_2_. Metal oxide semiconductor (MOS) gas sensors have been investigated for gas sensing detection of toxic and flammable gases [6,7,8]. 

Among the MOS gas sensors, ZnO and SnO_2_ as n-type metal oxide semiconductors (MOSs) have attracted much attention due to their excellent electrical properties and stable chemical and thermal features [9,10,11]. Moreover, other n-type MOSs, such as Fe_2_O_3_ [12], WO_3_ [13], In_2_O_3_ [14] and TiO_2_ [15], and p-type MOS such as NiO [16], Co_3_O_4_ [17], Cr_2_O_3_ [18], CuO [19] and Mn_3_O_4_ [20] have been extensively studied. As a typical n-type MOS with wide band gap energy of 3.37 eV at room temperature [21,22], ZnO has been widely employed because of its unique sensing properties and widespread applications [23]. In terms of performance, the most important indexes of ZnO-based gas sensors are selectivity, sensitivity, response-recovery speed (time) and stability (marked as “4S”). Unfortunately, the main disadvantage of ZnO-based gas sensors is the poor selectivity [24]; it is difficult for the ZnO-based gas sensors to differentiate the aforementioned two gases because of their similar behaviors. 

Some strategies have been employed to overcome the low selectivity of this kind of gas sensor, including regulation of the optimal operating temperature [25,26,27]; doping or loading of a noble metal [28,29,30,31,32]; modification of chemical composition, morphology and microstructure of the sensor materials [33,34,35]; and construction of n–n [36,37,38,39] or p–n junction [40,41,42,43,44,45,46,47]. Among them, the construction of p–n junction is expected to be a promising strategy due to the fact that the active sites of the p–n composites are different for the target gases. However, although the discriminability of gas sensors based on ZnO has been enhanced, these sensors could not precisely distinct the homogenous gases. Thus, poor discriminability of gas sensors for homogenous gases is still a challenge which limits the improvement in the performance of this type of gas sensor.

In this paper, ZnO–*x*NiO composites were designed and synthesized via a facile sol–gel process, and their gas sensing performances were systematically studied. We illustrated that the poor discriminability of gas sensors based on ZnO against CO and H_2_ can be enhanced via changing the amounts of the p or n-type oxide materials in p–n composites. The results of gas sensing properties of ZnO–*x*NiO sensors indicated that the ZnO–0.425NiO sensor sintered at 550 °C and operated at 350 °C showed n-type response for H_2_ and p-type response for CO; the special behavior of the opposite gas sensing responses of the ZnO–0.425NiO composite to CO and H_2_ suggests that the ZnO–0.425NiO composite can accurately differentiate individual CO and H_2_. It is worth noting that ZnO–0.425NiO composite can accurately identify whether the gas is CO or H_2_, rather than selectively detecting the mixture of CO and H_2_. Hence, we propose an effective method by adjusting the components of the composites to solve the poor discriminability problem of the ZnO-based sensors so that they can accurately distinguish CO and H_2_. Furthermore, the phenomenon of the abnormal sensing behavior of ZnO–0.425NiO for H_2_ and CO is explained in detail.

## 2. Experimental Details

### 2.1. Chemicals

Zinc acetate dehydrate (Zn(CH_3_COO)_2_·2H_2_O), nickel chloride hexahydrate (NiCl_2_·6H_2_O), polyvinyl pyrrolidone (PVP, K-30), absolute methanol (CH_3_OH), ethyl cellulose (M70) and α-terpineol (C_10_H_18_O) were purchased from Sinopharm Chemical Reagent Co. LTD (Beijing, China). All the reagents were of analytical grade and used directly without further purification.

### 2.2. Preparation of Materials

ZnO–*x*NiO composites with different molar ratios of Zn/Ni were prepared using a sol–gel method [48,49] in the following three steps. (a) 1 g of Zn(CH_3_COO)_2_·2H_2_O was dissolved into a mixture of 5 mL distilled water and 10 ml methanol to form a solution; then, 0.1 g of PVP was added into the above solution and stirred until all PVP were completely dissolved. Next, the solution was heated at 75 °C for 1 h until the ZnO gel formed. (b) NiCl_2_·6H_2_O was added into the ZnO gel and the mixture was stirred to form a homogeneous solution, which was further dried at 150 °C for 1 h at ambient condition. (c) The product was grounded for 10 min to obtain the ZnO–*x*NiO powder, which was further annealed at 550 °C in air atmosphere for 3 h. For convenience, the composites with a molar ratio of Zn:Ni = 1:*x* were named ZnO–*x*NiO (*x* = 0.111, 0.350, 0.400, 0.425, 0.429, 0.450, 0.667, 1.000), respectively. Pristine ZnO was prepared using the same steps, but without the addition of NiCl_2_·6H_2_O in the second step. Additionally, ZnO–0.425NiO was annealed at 500, 550 and 600 °C to explore the influence of sintering temperature on gas sensing properties.

### 2.3. Fabrication of Gas Sensors

**Figure 1:**
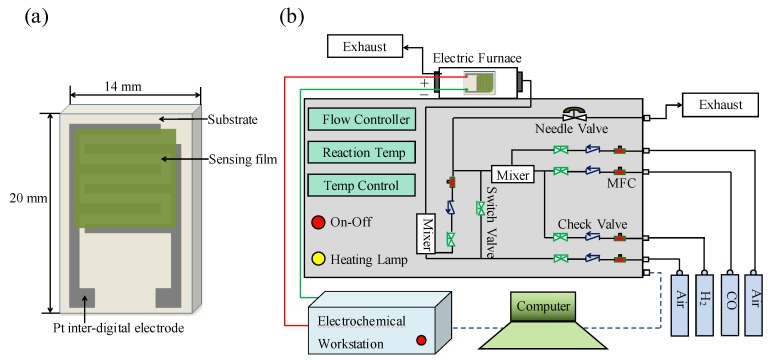
Schematic diagram of (**a**) the sensor component and (**b**) the gas sensing measurement system.

ZnO–*x*NiO gas sensors were prepared by printing ZnO–*x*NiO composites and platinum (Pt) on aluminum oxide substrate as the sensing materials and digital electrode, respectively. Silk screen printing was employed to fabricate the gas sensors as follows. (a) The aluminum oxide substrate was printed with Pt electrodes, and then roasted at 800 °C for 3 h in muffle furnace; (b) 1 g of ethyl cellulose was added into 10 mL of α-terpineol, and then the mixture was stirred and heated at 80 °C for 1 h to prepare the binder; (c) 1 g of ZnO–*x*NiO composite and 0.1 g of the binder were mixed and ground for 20 min to obtain the paste; (d) the paste was printed on the alumina substrate with digital electrodes using silk screen printing, and then the fabricated sensor was annealed at 550 °C. Figure 1a depicts a typical image of the fabricated sensor. 

### 2.4. Gas Sensing Measurements

Figure 1b depicts a typical image of the gas sensing system which consists of three main parts: an electric furnace, an electrochemical workstation (CHI660E, Chenhua Instruments Inc, Changsha, China) and a gas distribution system with a mass flow device. The specific process for the analysis of gas sensing performance is as follows. (a) The sensor was placed in an electric furnace and the two electrodes from the sensor were connected to the electrodes of the electrochemical workstation through platinum wire. (b) Air was used as carrier gas; the gas mass flow meter was used to monitor gas flow; the total flow rate through the electric furnace was maintained at 500 mL/min. (c) A constant voltage of 5 V was applied to the sensor and the I-t curve was obtained by means of an electrochemical workstation, the resistance was calculated based on Ohm’s law (R = V/I). In this paper, “Ra” stands for the resistance of the gas sensor in air, and “Rg” stands for the resistance of the gas sensor in the presence of target gas. The response of a sensor was defined as response (R) = Ra/Rg − 1.

### 2.5. Material Characterizations

The crystal structures of ZnO and ZnO–*x*NiO composites were studied using X-ray diffraction (XRD, X’Pert Pro, PANalytical B.V., Almelo, The Netherlands), using copper Kα1 radiation (λ = 1.542 Å); the scanning range was 10–90° and the scanning speed was 10°/min. The compositional and surface physico-chemical states of ZnO–0.425NiO were further characterized. The microstructure of ZnO–0.425NiO was studied by means of high resolution transmission electron microscopy (HRTEM, JEM-2100F, Tokyo, Japan), and energy dispersive X-ray spectroscopy (EDS). The surface physico-chemical states of ZnO and ZnO–0.425NiO were researched using X-ray photoelectron spectroscopy (XPS, ThermoFisher, Waltham, MA, USA) with Al Kα radiation. The phase identifications of ZnO-0.425NiO composite and pristine ZnO were confirmed by means of Raman spectrometer (Raman, DXR2, ThermoFisher, Waltham, MA, USA) with a laser wavelength of 514 nm under ambient conditions. The photoluminescence spectra (PL, HITACHI, Tokyo, Japan) were used with the Xe laser of 325 nm to study the recombination of electrons-holes at the ambient condition.

## 3. Results and Discussion

### 3.1. Structural and Morphological Characterization

**Figure 2:**
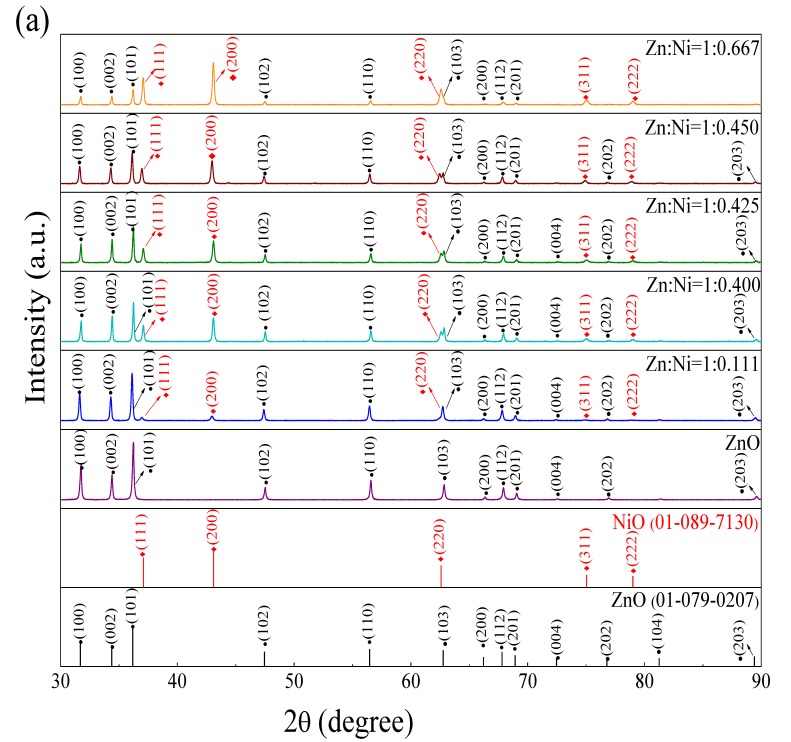

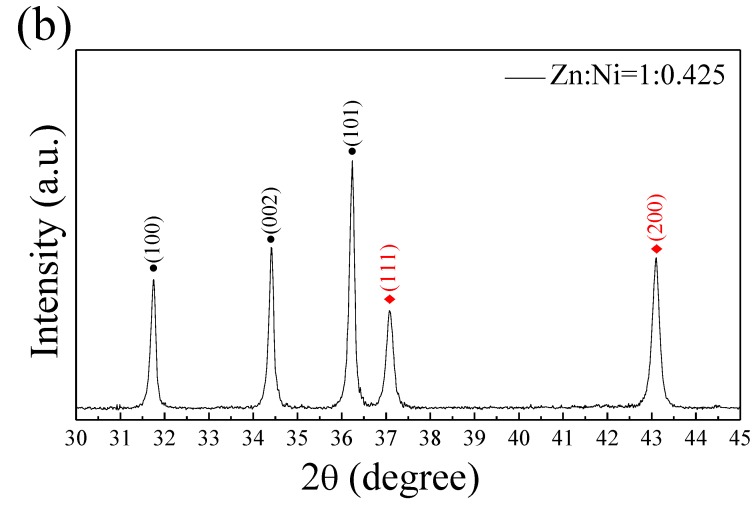
(**a**) XRD patterns of pristine ZnO and ZnO–*x*NiO composites annealed at 550 °C with different molar ratios of Zn:Ni. (**b**) Partially enlarged XRD patterns of ZnO–0.425NiO composite annealed at 550 °C.

XRD of ZnO and ZnO–*x*NiO composites annealed at 550 °C were carried out to illustrate the crystal structure of ZnO and the influence of Ni doping on the crystal structure ZnO. As shown in Figure 2a, all diffraction peaks can be indexed as individual ZnO and NiO. The diffraction peaks located at about 2θ = 37.09°, 43.09°, 62.59°, 75.05° and 79.02° are corresponding to the (111), (200), (220), (311) and (222) lattice planes of the cubic NiO (reference code: 01-089-7130, a = b = c = 4.1944 Å, space group Fm-3m), respectively. Diffraction peaks centered at 2θ= 31.69°, 34.38°, 36.18°, 47.46°, 56.46°, 62.76°, 66.22°, 67.81°, 68.92°, 72.47°, 76.79°, 81.26° and 89.42° correspond to the (100), (002), (101), (102), (110), (103), (200), (112), (201), (004), (202), (104) and (203) Miller indexes of hexagonal ZnO (reference code:01-079-0207, a = b = 3.2568 Å, c = 5.2125 Å, space group P63mc), respectively.

The grain sizes of ZnO and NiO in ZnO–0.425NiO were evaluated using theoretical Debye–Scherer equation (Equation (1)).

(1) $$d\;=\frac{0.89\lambda }{\beta \cos\theta }$$

where the wavelength λ of X-ray was 0.1542 nm; θ is the Bragg diffraction angle (θ); and β is full width at half maximum, which was calculated based on the most intense peaks of ZnO and NiO in Figure 2b. The peaks of (100), (002) and (101) planes for ZnO and the peaks of (111) and (200) planes for NiO were considered to evaluate the crystal structure factors, as summarized in Table 1. The average grain sizes of ZnO and NiO in ZnO–0.425NiO composite were obtained as 63.42 and 44.61 nm, respectively, which illustrate the formation of nanostructured materials.

In the XRD patterns, there are only diffraction peaks of individual ZnO and NiO; no unwished peaks of ternary phases associated with Ni and Zn-based were observed. In addition, compared with the XRD pattern of pristine ZnO, the center of each diffraction peak in the ZnO–*x*NiO composite was not shifted, which indicates that hexagonal ZnO and cubic NiO crystals are formed after sintering. Moreover, the n-ZnO/p-NiO heterojunctions form at the interface of the grains without changing the crystal structure of ZnO. Thus, the effect of variation in the crystal structure of ZnO on sensor response should be eliminated and we only focus to the influence of p–n heterojunctions on the gas sensing properties. Moreover, ZnO and ZnO–*x*NiO composites with high purity and high-crystallinity have been successfully prepared. 

The intensity of diffraction peaks in the XRD spectra of ZnO decreases, gradually while the intensity of diffraction peaks of NiO increases gradually upon an increase in the molar ratio of nickel, which indicates that ZnO–*x*NiO composites with different nickel contents were obtained by regulating the concentration of n-/p-type semiconductors. 

**Figure 3:**
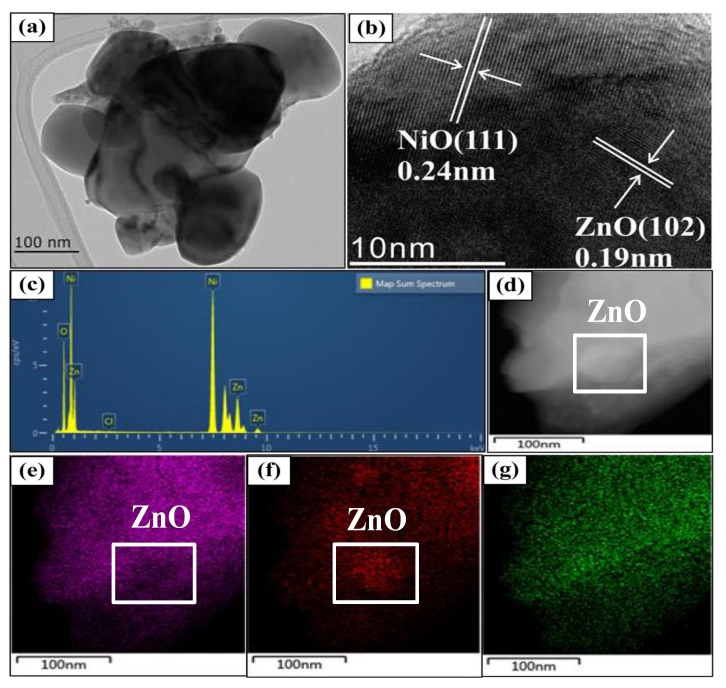
(**a**) TEM; (**b**) HRTEM; (**c**) EDS; (**d**) TEM-EDS dark-mapping scan; (**e**–**g**) TEM-EDS Ni, Zn and O color-mapping scans of ZnO–0.425NiO annealed at 550 °C.

Therefore, the obtained XRD results of ZnO–*x*NiO p–n junction with various Ni contents support the following discussion for the gas sensing phenomenon of ZnO–*x*NiO composite that strongly interferes with the target gasses. The microstructure and the elemental composition of the ZnO–0.425NiO composite annealed at 550 °C were further confirmed by TEM, HRTEM and EDS analysis. Figure 3a depicts the TEM image of ZnO–0.425NiO composite. It can be conspicuously seen that ZnO and NiO particles with uneven sizes are randomly scattered in the sample. 

The TEM images demonstrated many nanograins of ZnO and NiO in the composite that are either overlapped or in the close contact with each other. This is strong evidence for the development of p–n heterojunction in the ZnO–0.425NiO. Consequently, the results of the structural properties of ZnO–0.425NiO are inconsistent with the TEM results, suggesting that nanometer ZnO and NiO exist separately in the composite, and the p–n heterostructures of ZnO–NiO are developed at the interface of ZnO and NiO. Figure 3b portrays the HRTEM image of ZnO–0.425NiO composite. The crystal plane spacings of 0.24 and 0.19 nm are attributed to the crystal plane spacings of NiO (111) and ZnO (102), respectively. Moreover, the crystal lattice fringe is clear, and the acquired image exhibits a high crystallinity, which is consistent with the obtained XRD results. 

Figure 3c illustrates the EDS spectrum of ZnO–0.425NiO composite and the spectrum reveals the presence of Zn, Ni and O elements in ZnO–0.425NiO sample; there is no Cl element in the composite, which further confirms the presence of Ni in the form of NiO. Figure 3d–g shows the EDS dark and color mapping scan images of ZnO–0.425NiO, and Figure 3e–g shows Ni, Zn, and O elemental color-mapping scans, respectively. Figure 3d–g evidently demonstrates the presence of O, Zn and Ni elements in the samples, which confirms the existence of ZnO nanoparticles among the NiO nanoparticles. Combined with the XRD patterns and TEM images, the results further indicate that individual ZnO and NiO particles are randomly dispersed into the composites. This is another powerful piece of evidence to illustrate the formation of the p–n heterojunction, which is additional proof for the gas sensing phenomenon.

**Figure 4:**
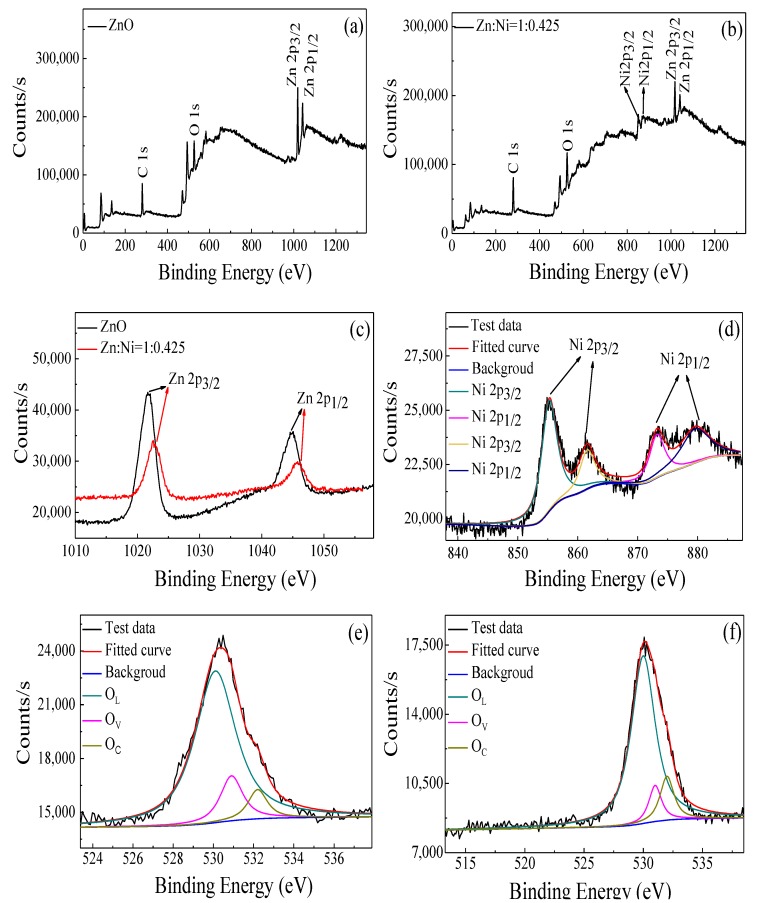
XPS survey spectra of ZnO and ZnO–0.425NiO annealed at 550 °C: (**a**) full spectra of ZnO, (**b**) full spectra of ZnO–0.425NiO, (**c**) Zn 2p spectra of ZnO and ZnO–0.425NiO, (**d**) Ni 2p spectra of ZnO–0.425NiO, (**e**) O 1s spectra of ZnO, (**f**) O 1s spectra of ZnO–0.425NiO.

XPS spectra of ZnO and ZnO–0.425NiO composite annealed at 550 °C were acquired to demonstrate the surface elemental analysis of pristine ZnO and ZnO doped with Ni. Figure 4a,b delineates the XPS full spectra of ZnO and ZnO–0.425NiO composite. The C1s peak located at 284.6 eV was used to calibrate the XPS spectra. The peaks of Zn 2p, Ni 2p and O 1s appear in the XPS spectrum of ZnO–0.425NiO composite, which is consistent with the results obtained from EDS spectra. Figure 4c shows the high-resolution XPS spectra of Zn 2p state in ZnO and ZnO–0.425NiO. The peaks of Zn 2p_3/2_ and Zn 2p_1/2_ states for ZnO are located at 1021.69 and 1044.62 eV; the peaks of Zn 2p_3/2_ and Zn 2p_1/2_ for ZnO–0.425NiO composite are centered at 1022.67 and 1045.6 eV; the energy difference between Zn 2p_3/2_ and Zn 2p_1/2_ is about 23 eV both for ZnO and ZnO–0.425NiO specimens, which are attributed to a normal state of Zn^2+^ in ZnO [50,51]. 

Compared with that of ZnO, the peaks of Zn 2p_1/2_ and Zn 2p_3/2_ for ZnO–0.425NiO composite are shifted to higher binding energy by 0.98 eV, and the reason for this is higher electronegativity of Ni (χ = 1.91) with respect to Zn (χ = 1.65) [52,53,54]. To put it differently, the discrepancy in the electronegativity of Ni and Zn resulted in attraction of Zn electrons by Ni; therefore, the density of outer valence electrons and the screening effect are reduced, which in turn improve the attraction of nuclear and binding energies of the inner electrons [55]. Figure 4d delineates the high-resolution XPS spectrum of the Ni 2p peaks for ZnO–0.425NiO composite. The peaks of Ni 2p_3/2_ and its satellite peaks are located at 855.23 and 861.59 eV; the peaks of Ni 2p_1/2_ and its satellite peaks are centered at 873.22 and 879.58 eV, respectively. The energy difference between the Ni 2p_3/2_ and Ni 2p_1/2_ is about 18 eV, which is in good agreement with the previously reported values for Ni^2+^ in NiO [56]. 

The XPS results are consistent with those of XRD and TEM, which prove the presence of ZnO and NiO in ZnO–0.425NiO. It is evidently known that the reaction of gas on the surface of the sensor results in the electron transfer on the surface which is used to evaluate the gas sensing properties. The size of the particles, the oxygen adsorption and the surface physico-chemical state are three crucial factors for the evaluation of gas sensing performance. Figure 4e,f shows the high-resolution XPS spectra of the O 1s state of ZnO and ZnO–0.425NiO composite. The O 1s broad peak is separated into three asymmetric peaks centered at 530 ± 0.2, 531 ± 0.2 and 532 ± 0.2 eV for ZnO and ZnO–0.425NiO, which correspond to lattice oxygen, vacancy oxygen and chemisorbed oxygen, respectively [57]. 

**Figure 5:**
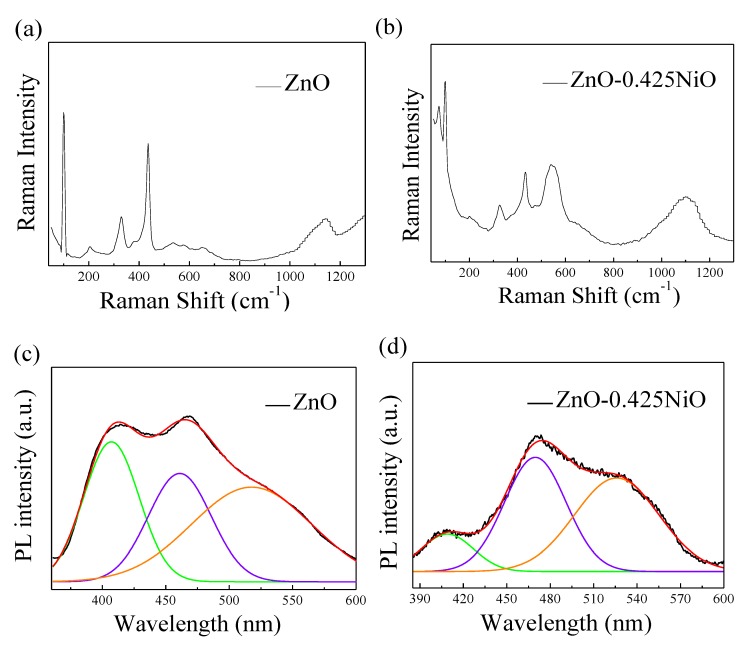
Raman spectra of the (**a**) pristine ZnO and (**b**) ZnO–0.425NiO composite annealed at 550 °C; photoluminescence (PL) spectra of (**c**) pristine ZnO and (**d**) ZnO–0.425NiO composite annealed at 550 °C.

The Raman spectra of pristine ZnO and ZnO–0.425NiO composite were obtained. The peaks in Figure 5a correspond to the characteristic peaks of ZnO [58]. The Raman spectrum of ZnO–0.425NiO composite which is shown in Figure 5b is different from the spectrum of pristine ZnO, which is shown in Figure 5a; the reason for this change may be the contribution of NiO. Figure 5c,d displays the photoluminescence (PL) spectra of pristine ZnO and ZnO–0.425NiO composite annealed at 550 °C with excitation wavelength of 325 nm and carried out at ambient condition. ZnO and ZnO–0.425NiO composite both exhibit peaks at around 410, 465 and 526 nm [59]. The emission peak located at about 410 nm and the peak located at around 465 nm are attributed to Zinc vacancy (Vzn) and intrinsic defects, respectively. The peak located at about 526 nm is attributed to the transition between the electrons near the conduction band and the deeply trapped holes [60].

### 3.2. Sensing Properties of ZnO–xNiO

**Figure 6:**
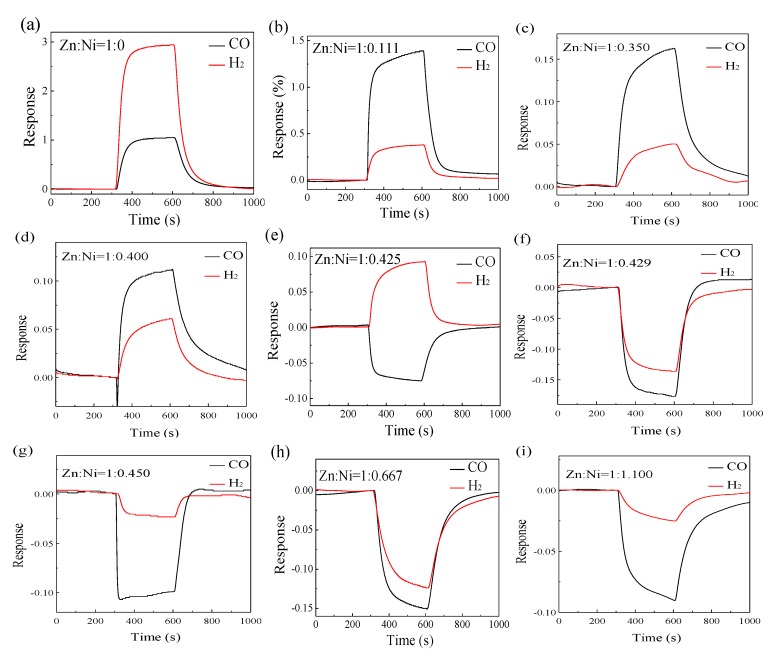
Responses of ZnO (**a**) and ZnO–*x*NiO (*x* = 0.111, 0.350, 0.400, 0.425, 0.429, 0.450, 0.667, 1.100) (**b**–**i**) annealed at 550 °C to 400 ppm CO and H_2_ at the operating temperature of 350 °C.

ZnO and the ZnO–*x*NiO composites annealed at 550 °C were exposed to 400 ppm CO and H_2_ at the operating temperature of 350 °C, and the results are demonstrated in Figure 6a–i. It is commonly known that when an n-type MOS is exposed to reducing gases, the resistance decreases, and the response is positive based on the aforementioned theoretical expression (R = Ra/Rg-1). When p-type MOS is exposed to reducing gases, the resistance increases and the response value is negative. It is evident from Figure 6 that the gas sensing responses to H_2_ and CO show an n-type to p-type transformation upon an increase in the value of “*x*” in ZnO–*x*NiO composite. The gas sensing behavior of ZnO–*x*NiO composite to CO is transformed from n-type to p-type with the critical “*x*” value between 0.4 and 0.425, while the critical “*x*” value for this transformation to H_2_ is between 0.425 and 0.429. As a result, ZnO and the ZnO–*x*NiO gas sensors with *x* < 0.425 show an n-type response to both CO and H_2_ gases, whereas ZnO–*x*NiO sensors with *x* > 0.425 show a p-type response to these two gases. 

However, the sensing results illustrate that the gas sensors based on ZnO–0.425NiO exhibit an n-type response to H_2_ and an opposite response (p-type) response to CO. Consequently, the sensing behavior (p-type or n-type) of ZnO–*x*NiO gas sensors depends on the composition of Zn:Ni, and their responses transformed from n-type to p-type with different critical values of “*x*” in ZnO–0.425NiO. Hence, an ultra-high gas sensing discriminability can be obtained for the gas sensors by adjusting the concentration of the composites. Apart from the obtained experimental results, the following explanation of this special gas sensing behavior is proposed. The electrical conductivity of ZnO–*x*NiO with *x* < 0.425 is dominated by ZnO-ZnO homojunctions, so the gas sensing behavior obeys to the depletion layer models. In contrast, the electrical conductivity of ZnO–*x*NiO with *x* > 0.425 is dominated by NiO–NiO homojunctions, so the gas sensing behavior obeys to the accumulation layer models [61]. 

The electrical conductivity of ZnO–0.425NiO is dominated by ZnO-NiO heterojunctions and the connection of ZnO and NiO results in the electrons flowing from n-type ZnO to p-type NiO to reach the same Fermi energy level. Simultaneously, the heterojunctions form at the interfaces between ZnO and NiO and a potential barrier is formed. As soon as the reducing gas is adsorbed on the surface of ZnO–0.425NiO, the gas supplies electrons to the surface of the sensor and the electrons transfer back into the conduction band of ZnO and NiO. 

In this case, the potential barrier height of both ZnO and NiO decrease and the thickness of electron depletion layer and vacuum accumulation layer becomes thinner; hence, the electron concentration of ZnO increases and the hole concentration of NiO decreases. The abnormal sensing behavior of ZnO–0.425NiO can be attributed to various adsorbability of CO and H_2_ on the surface of ZnO and NiO that result in discrepancy of content changes between the electron concentration increase on the surface of ZnO and the hole concentration decrease on the surface of NiO. As delineated in Figure 6a, the gas sensing response of pristine ZnO to 400 ppm H_2_ is higher than that of CO, which originated from the fact that H_2_ prefers to adsorb on the surface of ZnO. 

When ZnO–0.425NiO is exposed to H_2_, the increase of the electron concentration in ZnO is much more than the decrease of hole concentration in NiO, and therefore, the decreased potential barrier height of ZnO is greater than that of NiO. Thus, in presence of H_2_, the sensing properties of ZnO–0.425NiO are dominated by the variation of electron concentration and the sensor is electrically conductive and in this case the carrier is the electron. Therefore, the gas sensing behavior of ZnO–0.425NiO composite exhibits an n-type response to H_2_. In contrast, when the NiO is doped into the ZnO material, as shown in Figure 6b, the gas sensing response of ZnO –0.111NiO to 400 ppm CO is higher than that to 400 ppm H_2_, which can be explained, as the CO prefers to adsorb on NiO of ZnO–0.111NiO. 

To put it differently, when CO is introduced, the decrease of hole concentration in NiO is much more than the increase of electron concentration in ZnO, and therefore, the decrease of potential barrier height of NiO is greater than that of ZnO. When CO gas exists, the sensing properties of ZnO–0.425NiO are dominated by the variation of hole concentration, and the sensor is hole conductive; and in this case, the carrier is the hole. Therefore, the gas sensing behavior of ZnO–0.425NiO composite illustrates a p-type response to CO. An intriguing gas sensing response to CO and H_2_ have been obtained as illustrated in Figure 6. It is evident that the gas sensing response of ZnO–0.425NiO is lower than that of pristine ZnO, and the reason for this presence of more p–n heterojunctions in the ZnO–0.425NiO composite and the formation of a potential barrier at the interface of the ZnO and NiO in the ZnO–0.425NiO, are that this barrier can inhibit the flow of electrons and decrease the gas sensing response. 

**Figure 7:**
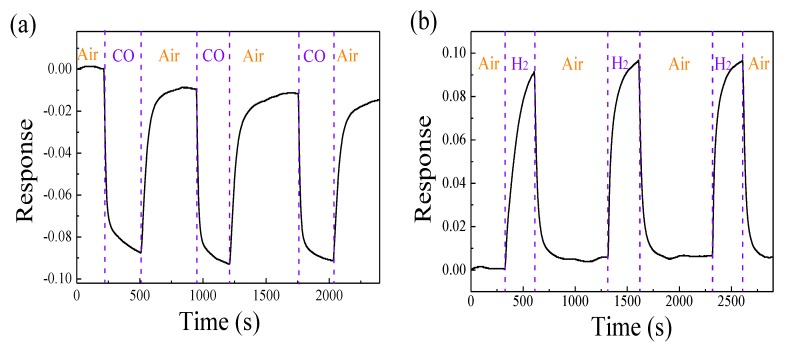
Three cycles of dynamic gas sensing response curve of ZnO–0.425NiO annealed at 550 °C to 400 ppm CO (**a**) and H_2_ (**b**) at 350 °C.

The sensing reproducibility and stability of ZnO–0.425NiO to CO and H_2_ were analyzed. Figure 7a,b depicts three cycles of gas sensing response curves of ZnO–0.425NiO annealed at 550 °C to 400 ppm (a) CO and (b) H_2_ at the operating temperature of 350 °C, respectively. The results indicate the reproducibility and stability of such behavior. It is worth noting that the process conditions for reproducibility of such results requires a high accuracy, and when any of the factors affecting gas sensing behavior (p-type or n-type), including concentration of sensing materials, operating temperature, etc., changes, then the critical value *x* for the p–n transformation might be changed. In addition, the heating mode of the sensor which is heating the substrate [62] or the whole gas sensing detection chamber [63] may also affect the gas sensing behavior. 

**Figure 8:**
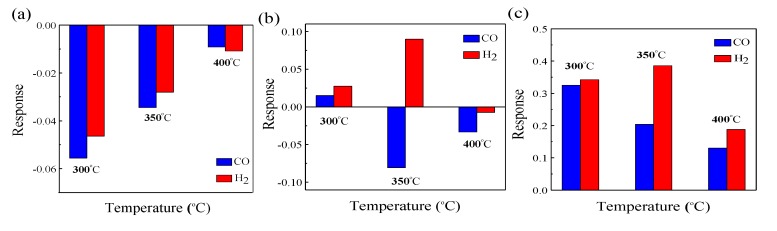
Response value of ZnO–0.425NiO annealed at (**a**) 500 °C, (**b**) 550 °C, (**c**) 600 °C to 400 ppm CO at different operating temperatures.

As shown in Figure 8a–c, ZnO–0.425NiO gas sensors annealed at 500, 550 and 600 °C were exposed to 400 ppm CO and H_2_ at 300, 350 and 400 °C operating temperatures, respectively. Among these different cases, the gas sensor based on ZnO–0.425NiO as the sensing material and annealed at 500 °C delineates a p-type response to both CO and H_2_, while the same gas sensor annealed at 600 °C demonstrates an n-type response to these two gases.

Surprisingly, the gas sensor based on ZnO–0.425NiO composite and annealed at 550 °C showed an n-type response to CO and H_2_ at 300 °C and showed a p-type response to CO and H_2_ at 400 °C, while it showed an n-type response to H_2_ and a p-type response to CO at 350 °C (Figure 8b). Therefore, in addition to the molar ratio of Zn:Ni in the composite materials, the heat treatment including annealing and operating temperatures have fundamental roles in the response (p or n-type) of gas sensors. Therefore, the sensing behavior of ZnO–*x*NiO gas sensors is influenced by factors such as the contents of sensing materials and their compositions, and calcination and working temperatures. As a result, it was found that ZnO–0.425NiO sensor annealed at 550 °C showed opposite gas sensing behavior to CO and H_2_ at 350 °C operating temperature.

## 4. Conclusions

ZnO–*x*NiO composites were successfully prepared using simple sol–gel method. They were annealed at different temperatures and used as the sensing materials for the fabrication of gas sensors. The sensitivity results elucidate that the response of ZnO–0.425NiO could be of p-type or n-type, depending on factors such as the molar ratio of Zn/Ni and heat treatment, including post-annealing and operating temperatures. The obtained gas sensing results evidently illustrate that ZnO–0.425NiO annealed at 550 °C and tested at 350 °C exhibited a p-type response to CO but the opposite response (n-type) to H_2_, which indicates the modulation of molar ratio of Zn/Ni in the p–n composites (ZnO–*x*NiO) could be an effective way to improve the poor discriminability of this type of gas sensor to the homogeneous gases. The explanation of the special gas sensing behavior of ZnO–0.425NiO to CO and H_2_ is proposed based on band theory and the observed experimental observations including XRD, HRTEM and XPS. This paper provides an idea for designing p–n composites to improve the gas sensing discriminability of target gases.

## Figures and Tables

**Table 1:** Parameters for the calculation of crystallite sizes.

Metal Oxide	Diffraction Planes	Diffraction Angles (deg.)	FWHM (β)	Crystallite Size (nm)	Average Size (nm)
ZnO	(100)	31.69	0.1314	62.24	63.42
	(002)	34.38	0.1248	65.99	
	(101)	36.18	0.1334	62.04	
NiO	(111)	37.09	0.1877	44.21	44.61
	(200)	43.09	0.1879	45.01	
